# Structural Mechanisms of Hexameric Helicase Loading, Assembly, and Unwinding

**DOI:** 10.12688/f1000research.7509.1

**Published:** 2016-01-27

**Authors:** Michael A. Trakselis

**Affiliations:** 1 Department of Chemistry and Biochemistry, Baylor University, Waco, Texas, 76798, USA

**Keywords:** Hexameric Helicase, DNA replication, DNA unwinding, Helicase Loading, Helicase Assembly

## Abstract

Hexameric helicases control both the initiation and the elongation phase of DNA replication. The toroidal structure of these enzymes provides an inherent challenge in the opening and loading onto DNA at origins, as well as the conformational changes required to exclude one strand from the central channel and activate DNA unwinding. Recently, high-resolution structures have not only revealed the architecture of various hexameric helicases but also detailed the interactions of DNA within the central channel, as well as conformational changes that occur during loading. This structural information coupled with advanced biochemical reconstitutions and biophysical methods have transformed our understanding of the dynamics of both the helicase structure and the DNA interactions required for efficient unwinding at the replisome.

## Introduction and context

Cell proliferation relies on the exact replication of an organism’s genetic material in a rapid but precisely controlled and efficient manner. The process and mechanism of DNA replication directs targeted and repetitive enzymatic activities towards long linear polymers of DNA. Interestingly, organisms have evolved a number of toroidal DNA replication and repair protein complexes that can maintain repetitive catalysis by encircling the DNA substrate. These protein-DNA rotaxane-like systems have the intrinsic ability to be processive enzymes due to their topological linkage with the substrate. As such, they provide inherent challenges to the loading and encircling of DNA. The steps required for the loading and encircling of circular protein complexes onto DNA provides for a higher level of regulation, which is required to restrict cell cycle progression and control DNA replication initiation. Because of this, the most highly regulated component within the DNA replisome is the loading and activation of the hexameric helicase, which dictates both the initiation steps and the elongation rate of DNA replication. Even though the general toroidal hexameric helicase structure has been known for more than two decades, the mechanisms for loading, encircling, activating, and unwinding are only just being discovered. These recent advances have been primarily aided by higher resolution structures that include DNA, better mechanistic descriptions of the interactions of the helicase with each separated strand of single-stranded DNA (ssDNA), and higher order *in vitro* reconstitution of DNA replication systems. It is an exciting time to be a part of the hexameric helicase field as big questions regarding dynamic structure-function relationships with DNA are poised to be revealed.

## Hexameric helicase architectural conservation

**Figure 1.**
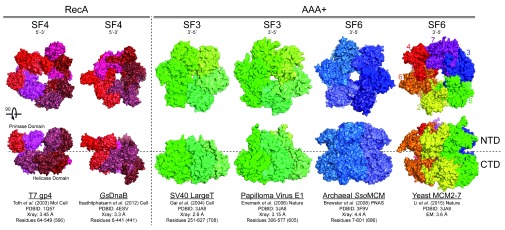
Structural conservation of hexameric helicases. Hexameric helicases are shown from different domains (RecA or AAA ^+^) and superfamilies (SFs) with associated unwinding polarity and references. View from the N-terminal domain (NTD) rotated 90° to visualize the lateral length from the C-terminal domain (CTD) to the NTD.

Although the general architecture of hexameric DNA replication helicases is shared across organismal domains, there is strong evidence that classes of these enzymes have evolved independently for a role in DNA replication ^1^. Although all hexameric helicases are members of the broader P-loop family of ATPases ^2^, individual evolution of RecA domains gave rise to the superfamily (SF) 4 helicases including T4 gp41, T7 gp4, bacterial DnaB, and mitochondrial Twinkle ^3^, while SF3, including SV40 Large T antigen (SV40 L-Tag) and papilloma virus E1, and SF6, including archaeal and eukaryotic minichromosome maintenance proteins (MCM), helicases came from an ATPases associated with a variety of cellular activities (AAA ^+^) clade ( Figure 1 & Table 1) ^4, 5^. Regardless of the origin, these systems have all converged on a common ring-shaped architecture wherein a central channel is used to repetitively engage and translocate along ssDNA during unwinding. Bacterial and phage SF4 helicases are perhaps the best studied and have contributed most to our understanding of DNA unwinding, but more recent emphases on SF3 and SF6 helicases are providing insight into structure-function relationships across SFs.

**Table 1.** Model Replication Helicase Loading and Activation Components.

	Initiator	Helicase	SF (Polarity)	Loader	Activator	Accessory
**Eukaryotes**						
*Sce*/ *Hsa*	Orc1-6	MCM2-7	SF6 (3’-5’)	Cdt1/Cdc6	CDK/DDK	GINS/Cdc45
**Archaea**						
*Sso*/ *Mth*	Orc1-2(3)	MCM	SF6 (3’-5’)	Cdt1	**-**	GINS/RecJ
**Bacteria**						
*Eco*/ *Bsu*	DnaA	DnaB	SF4 (5’-3’)	DnaC (DnaI)	**-**	Rep
**Mitochondria**						
*Hsa*	**-**	Twinkle	SF4 (5’-3’)	**-**	**-**	**-**
**Viral**						
papilloma E1	**-**	E1	SF3 (3’-5’)	**-**	**-**	**-**
polyoma SV40	**-**	Large T-ag	SF3 (3’-5’)	**-**	**-**	**-**
**Bacteriophage**						
T4	**-**	gp41	SF4 (5’-3’)	gp59	**-**	**-**
T7	**-**	gp4	SF4 (5’-3’)	**-**	**-**	**-**

Helicase activity requires the presence of both nucleotide triphosphates (NTPs) ^6^ and Mg ^2+^ for unwinding ^7– 9^. Although it is not known exactly how ATP hydrolysis directly drives DNA unwinding, it is likely to progress in a sequential manner, with each subunit driving conformational changes throughout the hexamer that contribute to unwinding polarity ^4, 10– 14^. RecA-like helicases (SF4) translocate along ssDNA in the 5’ → 3’ direction, while AAA ^+^ enzymes (SF3 and SF6) translocate in the 3’ → 5’ direction ^13, 15– 18^. The core ATP binding and hydrolysis domains consist of conserved RecA-like or AAA ^+^ folds that exist within a single subunit or between adjacent subunits that include “Walker A and B motifs” for ATP binding and hydrolysis and a basic “arginine finger” residue for nucleotide turnover and conformational coupling ^3, 19– 22^. Conserved β-hairpin structures in AAA ^+^ helicases contribute differentially to DNA binding and unwinding to direct DNA through the channel ^11, 16, 22– 26^, although additional interactions with DNA have also been detected on the exterior surface of helicases ^15, 27, 28^.

Several high-resolution X-ray and electron microscopy (EM) structures have been reported for the apo and nucleotide-bound forms of hexameric helicases (T7 gp4 ^29, 30^, DnaB ^31, 32^, Mito Twinkle ^33^, SV40 Large-T ^11, 34^, *Sulfolobus solfataricus* MCM [ *Sso*MCM] ^23, 35^, and *Saccharomyces cerevisiae* MCM [ *Sc*MCM2-7] ^36^). The global shared architecture of the ring-shaped helicases is generally composed of two tiers: an N-terminal DNA-binding domain (NTD) and a C-terminal AAA ^+^ or RecA motor domain (CTD) ( Figure 1). The orientation of most helicases on DNA places the CTD toward the duplex double-stranded DNA (dsDNA) region and the NTD outwards ^22, 37^. The exception is E1, where the orientation is reversed, placing the NTD toward the duplex ^38^. Thus, motor domains are often positioned close to the dsDNA duplex, which can leave the NTD regions free to bind, stabilize, or act on the resultant ssDNA.

Across species, hexameric helicase NTDs seem to have evolved differential functions. T7 gp4 can be expressed as either a 56 kDa helicase only form or as a full-length two-domain 65 kDa helicase-primase ^39^. The composition of the T7 gp4 helicase hexamer is thought to be a mixture of the two forms *in vivo,* controlling the number of primases present for faster replication and less pausing. Other SF4 helicases, including T4 gp41 and *Escherichia coli* DnaB, interact with a separately encoded primase at the NTD in an analogous configuration. In those cases, the composition and ratio of helicase to primase is <1:1, and more often recognized as 6:3 ^31, 40^. For DnaB, ATP binding by the motor domain can induce conformational changes within the NTD collar that can regulate partner protein (i.e. DnaC or DnaG) selection ^41^. As can be seen in Figure 1, increasing organismal complexity through the SF3 and SF6 helicases (from left to right) generally increases the size of the NTD to where they have evolved additional β-hairpins and zinc-finger motifs for more stabilized binding of the encircled strand and double hexamer formation ^42, 43^. The expanded NTD also provides a platform for control of activity through helicase accessory protein binding (in the case of Cdc45 and GINS ^44^) or activation through phosphorylation by either cyclin-dependent kinase (CDK) or Dbf4-dependent Cdc7 kinase (DDK) ^45– 47^.

## Helicase loading and the encircling of DNA

**Figure 2.**
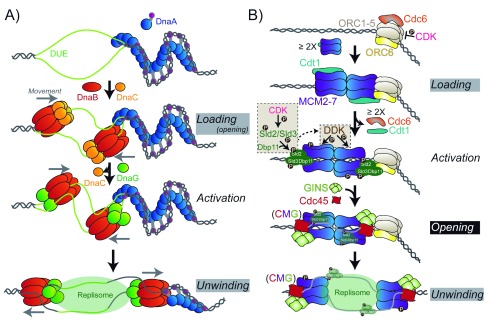
Assembly of Hexameric Replication Helicase at Origins. **A**) Loading of the bacterial DnaB helicase by the loader, DnaC, requires the destabilization of a DNA unwinding element (DUE) by the initiator protein DnaA. DnaB complexes with the primase, DnaG, to translocate along the lagging strand unwinding DNA ahead of the replication fork. **B**) Loading of the eukaryotic MCM2-7/Cdt1 complex requires the initial binding of the ORC complex (ORC1-6) and Cdc6. Interactions with Cdc6 and ORC with the CTD of MCM2-7 directs the adjacent loading of the first hexamer and dissociation of Cdc6 and Cdt1. Subsequent loading of the second hexamer is thought to proceed through direct interactions between the NTD of MCM2-7 to form the double hexamer. Activation of the helicase includes CDK phosphorylation of Sld2 and Sld3 to promote interaction with Dpb11 and stimulate DDK phosphorylation of MCM2/4/6 and recruitment of GINS and Cdc45 to form the CMG complex. Opening of the CMG complex and exclusion of the nontranslocating strand from the central channel activates unwinding and translocation on the leading strand. Gray and black boxes represent major and the foremost, respectively, queries remaining regarding structural conversions of helicases at origins.

The loading of hexameric helicases at replication origins and the associated steps required for encircling only one strand have been the subject of much debate over the years. What is clear is that the loading of the hexameric helicase generally requires the concerted action of accessory initiator proteins to locally melt duplex DNA and facilitate encircling of DNA. However, phage T7, mitochondrial Twinkle, and SV40 L-Tag helicases can load onto circular dsDNA on their own ^48– 50^. Within the three domains of life, the core ATPase activity and ordered assembly of replication initiation factors seem to be preserved to control the start of DNA synthesis ^51, 52^ ( Table 1). In bacteria, the initiator, DnaA, forms a multimeric right-hand filament at the replication origin, *oriC*, to induce unwinding or melting at an A-T rich DNA unwinding element (DUE) ( Figure 2A) ^53– 60^. Afterwards, the DnaB helicase is loaded on the top and bottom strands by concerted activities of DnaC and DnaA ^61– 66^. Once loaded, the primase, DnaG, interacts with DnaB, displaces DnaC ^40, 67^, and activates unwinding ^68^. The association of an accessory helicase, Rep, with DnaB may aid in replication fork progression ^69– 71^.

In archaea and eukaryotes, the binding of the origin recognition complex (ORC1-6) and Cdc6 to origins of replication is necessary for loading MCM2-7/Cdt1 complexes onto dsDNA to generate a pre-replication complex (Pre-RC) ( Figure 2B) ^72– 76^. The precise structural conformations and dynamics of MCM loading are not fully known, but the steps and components for assembly and activation of the eukaryotic MCM2–7 complex have been recently biochemically reconstituted *in vitro*, providing significant insight into the process ^77, 78^. In archaea, the homohexameric MCM complex exists as a closed ring in solution and would require initiation factors to stimulate opening into a helical conformation onto DNA ^79^. Alternatively, increases in temperature for these model hyperthermophilic archaeal MCMs may provide the thermal energy required for the destabilization of a subunit interface required for loading. The eukaryotic MCM2-7 helicase appears to be naturally open, with a labile 2-5 interface that can be trapped by ORC/Cdc6 ^80– 82^. The ORC1-6 complex is arranged in a two-layered cracked ring that encircles DNA and uses the helix-turn-helix domains to engage the MCM2-7 hexamer in a proposed ring-ring interaction ^83, 84^, in a manner similar to the loading mechanism of clamp/clamp-loader complexes onto dsDNA ^85^. The organization of the ORC complex also appears to be regulated and exists in either an autoinhibited ATP-bound form that precludes DNA binding or a proposed active form that requires a large conformational change in ORC1 that makes the complex competent for encircling DNA ^83^. Afterwards, the first MCM2-7 hexamer is loaded through direct interactions of MCM6-Cdt1 with the ORC1-6/Cdc6 complex ^86, 87^. The second MCM2-7 hexamer is loaded through contacts between the NTDs of the first loaded MCM2-7 hexamer, rather than through interactions with the ORC1-6/Cdc6 complex ^78, 84, 88, 89^. This generated the double hexamer complex, which has been known for years for MCM (and SV40 L-Tag) ^42, 90– 92^, but whether the double hexamer represents an active unwinding unit or an intermediate in the loading process was not known.

After loading of the MCMs, a series of steps are required to form the active unwinding complex ( Figure 2B). ATP hydrolysis by Cdc6 and ORC1 causes the dissociation of Cdc6 and Cdt1 ^93^. Subsequent phosphorylation of Sld2 and Sld3 by CDK promotes Dpb11 (DNA polymerase B-associated protein) to interact with MCM2-7 ^94– 96^. Phosphorylation of Sld3, in particular, recruits Cdc45 and GINS (Sld5, Psf1, Psf2, Psf3) to MCM2-7 and stimulates the DDK-dependent phosphorylation of MCM2 ^97^, as well as MCM4/6 ^98, 99^. These phosphorylation events allow opening of the MCM2/5 interface to extrude ssDNA that remains bound to Sld2/Sld3/Dpb11 ^100, 101^. The active unwinding CMG complex or “unwindosome” is formed through the association of Cdc45 and GINS with MCM2-7 at the labile MCM2-MCM5 interface ^80, 81^ along the waist between the NTD and the CTD ^44, 80, 102^. Cdc45 in particular blocks the MCM2-5 gate and prevents the loss of DNA from the central channel ^103^. In the reverse mechanism, Cdc45 may also be important for converting MCM2-7 encircled on dsDNA to encircling only a single DNA strand while excluding the other. Formation of the CMG complex widens the gap between the NTD and CTD at MCM2 and MCM5 while concomitantly narrowing the interface at the opposite MCM4 and MCM6 subunits. This induced spiral configuration may contribute to coupled ATP hydrolysis, propagating a conformational change through the MCM2-7 complex to translocate along and unwind duplex DNA ^44, 103^.

## Interactions with DNA: views of the encircled strand

**Figure 3.**
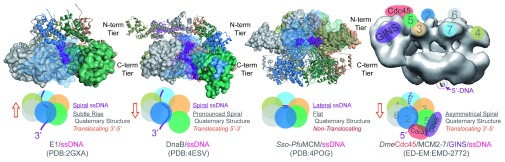
Conformational States of Hexameric Helicases Bound to the Encircled Strand. Space-filling representation of the C-terminal domain (E1 [SF3, 3’-5’] and DnaB [SF4, 5’-3’]) or N-terminal domain ( *Sso*- *Pfu* hybrid [SF6, 3’-5’]) interacting with the encircled strand (purple). Also shown is the electron microscopy structure of the *Drosophila* Cdc45/MCM2-7/GINS complex (CMG) (SF6, 3’-5’) with color-coded subunits. The conformational states of the active translocating hexamers representing rings with subtle rises (E1) or obvious spirals (DnaB and CMG) in the structures as well as helical single-stranded DNA (ssDNA) are indicated in the schematics. The flat *Sso*- *Pfu* hybrid structure represents a nontranslocating state used to identify a novel lateral DNA binding site. The orange box arrow indicates the translocation direction of the hexamer relative to the encircled ssDNA.

X-ray structures of hexameric helicases with oligonucleotides bound in the central channel (E1, *E. coli* Rho [ *Ec*Rho], *Bacillus stearothermophilus* [Bst] DnaB, and *Pyrococcus furiosus* MCM [ *Pfu*MCM]) have informed our understanding of the contacts and conformations required for translocation along ssDNA. In these co-crystal structures, ssDNA is bound in a helical conformation in the central channel making direct contacts with each subunit ( Figure 3). For E1 and Rho, the hexameric ring is proposed to remain closed, but conformational changes between subunits, coupled with sequential ATP binding and hydrolysis around the ring, direct ssDNA through the central channel through contact with DNA binding loops in a staircase motion ^13, 38, 104^. Each hexamer subunit interacts with one nucleotide of the oligo, predicating a step-size of one nucleotide per ATP hydrolyzed. This is consistent with the measured step-size of T7 gp4 of one base-pair unwound per dTTP hydrolyzed ^105^. For DnaB, the crystal structure resembles more of a lock washer, where similar conformational changes throughout the quaternary structure facilitate movement, with a step-size of two nucleotides per ATP hydrolyzed, maintaining a cracked ring structure ^106^. The ssDNA bound to the archaeal MCM seems to be trapped in a lateral orientation around the interior of the NTD, possibly identifying specific contacts during activation or unwinding, implying a step-size greater than one nucleotide per ATP hydrolyzed during translocation ^107^. The EM structure of the intact eukaryotic CMG complex bound to DNA is in a spiral or lock washer conformation ^44^, more similar to the DnaB/ssDNA complex. The crack in the ring between the MCM2 and 5 subunits is again held in check by the Cdc45 and GINS subunits and helps to stabilize the spiral configuration ( Figure 3). Of course, the impact and absolute degree of spiraling, wrapping, or compaction of the encircled strand will need to be validated experimentally, most likely using single-molecule approaches to measure end-to-end distances during loading and unwinding. Almost certainly the flat ring, the asymmetrical spiral, and the cracked lock washer structures represent intermediates during helicase activation and unwinding, but both conformations will also need to be validated further with additional high-resolution structural studies or rigorous biophysical characterizations to monitor the changes in the conformations.

## Interactions with DNA: Impact of the excluded strand

**Figure 4.**
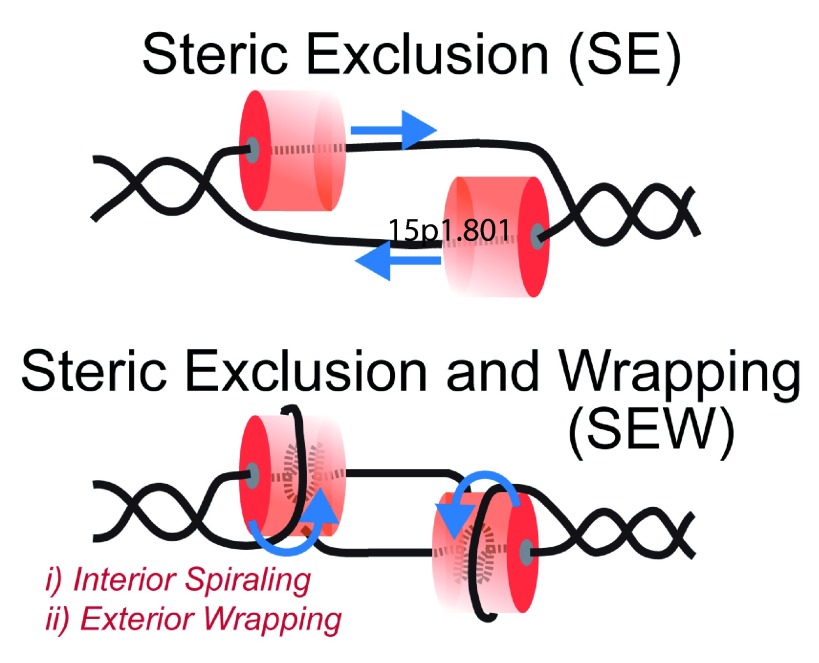
DNA Unwinding Models for Hexameric Replication Helicases. Steric exclusion (SE) model encircles the translocating strand and physically separates the nontranslocating strand outside of the central channel. The steric exclusion and wrapping (SEW) model takes into account specific interactions within the central channel (compaction or spiraling) as well as external interactions (binding or wrapping) with the excluded strand. Blue arrows indicate the direction of movement of the DNA strands with respect to the hexamer.

Based on the structures and associated biochemical data, the steric exclusion (SE) model, where one strand is encircled and the other is physically excluded, has become the consensus opinion for unwinding for hexameric replication helicases ( Figure 4) ^108^. One caveat to this model is that it generally ignores any contributions of the excluded strand to unwinding. Electrostatic interactions with the excluded strand on the external surface of hexameric helicases have been noted for archaeal MCM and shown to be important for unwinding, contributing to the development of the steric exclusion and wrapping (SEW) model ( Figure 4) ^17, 109^. Others have also noted that both ssDNA and dsDNA have a binding site on the external surface of other helicases ^15, 27, 28^. The dynamic and somewhat stochastic nature of unwinding has been attributed to interactions of ssDNA on the external surface of hexameric helicases E1 ^38^, T4 gp41 ^110^, and DnaB ^111^. In addition, subunits within the unwindosome complexes of SV40 L-Tag ^112^ and CMG ^113^ have been shown to interact with the excluded strand for loading and activation of unwinding. Intriguingly, DNA repair helicases have also been shown to sense damage or modifications on the excluded strand and stall unwinding ^114– 117^.

For SV40 L-Tag, initial binding to the origin may be directed by internal β-hairpins making direct contacts with the minor groove ^49^ and specific contacts of the origin binding domain (OBD) to the major groove ^118^. It is not currently understood how this initial dimer contact nucleates assembly of a double hexamer around dsDNA. Once loaded, SV40 L-Tag is proposed to convert from encircling duplex DNA to encircling ssDNA by pumping and extruding one strand out through side-channels ^11, 119^. Conformational changes within internal β-hairpins may direct the translocating strand through the central channel, while extruding the opposing strand. Using single-molecule experiments, researchers have shown that DNA unwinding proceeds with a single hexamer of L-Tag in a steric exclusion mechanism that is somewhat conformational mobile and able to bypass bulky adducts during translocation ^120^. In comparison, a novel mechanism has recently been proposed for E1 where duplex DNA enters the hexamer before being separated internally and forcing individual strands out through separate exits channels ^121^. Of course, the steps and dynamics for how these hexameric helicases convert from encircling duplex DNA to single strand separases by pumping DNA out through side-channels, opening of a gate, or through another unknown mechanism need to be visualized directly with high resolution. A recent EM structure shows the leading strand Pol ε ahead of the yeast CMG complex (at the CTD) and suggests a possible model where the encircled leading strand bends back and threads through a side-channel via the MCM2-5/Cdc45/GINS gate to enter the polymerase active site ^122^. Alternative models of replisome-DNA interactions were also proposed in this study.

With this emerging information, excluded or opposing strand interactions shown in the SEW model ( Figure 4) are poised to play multifaceted roles in loading, encircling, unwinding, and sensing of DNA. In the case of archaeal MCM, the external ssDNA binding path in the SEW model serpentines along the lateral length of the homohexamer, spanning the CTD and NTD, and even crossing and wrapping across multiple subunits (Graham & Trakselis, unpublished data) ^17^. The SEW model for archaeal MCM is analogous to a socket wrench, whereby the encircling of one strand represents the socket and external interactions with the excluded strand represent the directional ratchet controlling the speed and stabilization of unwinding. Whether the SEW model is conserved in all or most hexameric helicases and/or at what stages of helicase assembly it may occur remains to be determined. Currently, we have found that external interactions and dynamics with the excluded strand in the *E. coli* DnaB helicase are practically identical to that of *Sso*MCM, despite their opposing polarities (Carney & Trakselis, unpublished). On the other hand, for T7 gp4 the excluded ssDNA interacts with T7 DNA polymerase to generate a replisome complex, where the helicase and polymerases are within one nucleotide of the fork junction and the helicase can make no external contact with the excluded strand ^105, 123^. Next, it will be important to determine which of the eukaryotic MCM subunits (MCM2–7) interact specifically with the excluded strand or whether uniform binding sites have evolved on all subunits. It is intriguing that this external contact may help fill in some of the missing steps highlighted in the gray or black boxes depicted in Figure 2.

Although the structural features of the SEW model may be conserved with various hexameric helicases, both the mechanistic roles and molecular interaction sites may be different. In the case of *Sso*MCM, disruption of external interactions through mutagenesis reduced unwinding efficiency (3’-5’) ^17^, but analogous external mutations on DnaB show a stimulation in unwinding (5’-3’) (Carney & Trakselis, unpublished). Modification of the excluded strand to a morpholino oligo similarly stimulates the unwinding rate of T7 gp4 ^124^. Whether these effects result from opposite unwinding polarities or finely tuned control of unwinding rates and maintenance of the excluded strand requires further testing. However, detection and identification of these novel external interactions may provide a unique opportunity to target specific helicases for inhibition. As none of the different hexameric helicase families exhibit significant sequence homology outside of the center P-loop NTPase fold, novel exterior patches (e.g. between the CTD and the NTD) may provide idealized locations for specific targeting of small molecules that perturb unwinding through disruption of excluded strand contacts and avoid direct inhibition of the internal conserved ATPase site.

## Future directions

Although significant advances in our understanding of hexameric helicase assembly, loading, and unwinding have been made over the past few years from quantitative biophysical characterizations and various high-resolution structures, more work is required to reveal specific mechanistic steps and transitions. For example, the essential components for the initial loading of hexameric helicases onto DNA are well described, but the conformational changes that occur within the hexamers during the encircling of ssDNA are still unknown. After all these years, the black box in the whole mechanism is still the structural conversion of the helicase from encircling dsDNA to the encircled ssDNA directing the polarity of translocation and unwinding, primarily for SF3 and SF6 enzymes. Although much is known about the loading and activation mechanism in the Gram-negative *E. coli* system, far less is known about SF4 helicases in the Gram-positive organisms where DnaI acts as the loader ^65, 125, 126^ or in bacteria which lack DnaC/DnaI loader homologs altogether ^127, 128^.

Although there is a wealth of structural information on the static hexameric helicases themselves, there is still much debate on the mechanics of helicase action. No longer is the focus directly on the structure of the helicase protein itself. Instead, it has switched from identifying conformational changes, transacting proteins, and post-translational modifications that reveal how duplex DNA is destabilized and the path it takes to be excluded. Finally, although the unwinding mechanism of hexameric helicases was thought to be as simple as excluding one strand from the central channel, new information highlighting the specificity and importance of interactions with the nontranslocating strand have central implications on loading and unwinding mechanisms. It is these dynamic conformational steps from the viewpoint of both the helicase and the duplex DNA that will lead to the next transformational leap in replication helicase discovery.

## Abbreviations

AAA ^+^, ATPases associated with a variety of activities; ATP, adenosine triphosphate; Cdc6, cell division cycle 6; Cdc45, cell division cycle 45; CDK, cyclin-dependent kinase; Cdt1, chromatin licensing and DNA replication factor 1; CMG, Cdc45/MCM2-7/GINS complex; CTD, C-terminal domain; DDK, Dbf4-dependent Cdc7 kinase; Dpb11, DNA polymerase B-associated protein; dsDNA, double-stranded DNA; EM, electron microscopy; GINS, go-ichi-nii-san, Japanese for 5-1-2-3 for Sld5-Psf1-Psf2-Psf3; MCM, minichromosome maintenance proteins; NTD, N-terminal domain; NTPs, nucleotide triphosphates; ORC, origin recognition complex; Pre-RC, prereplication complex; Psf, partner with Sld5; SE, steric exclusion; SEW, steric exclusion and wrapping; SF, superfamily; Sld, synthetic lethal with dpb11; ssDNA, single-stranded DNA.
